# Pediatric Scoliosis Surgery—A Comprehensive Analysis of Treatment-Specific Variables and Trends in Latvia

**DOI:** 10.3390/medicina56040201

**Published:** 2020-04-24

**Authors:** Julian M. Rüwald, Janis Upenieks, Janis Ositis, Alexander Pycha, Yuval Avidan, Anna L. Rüwald, Robin L. Eymael, Frank A. Schildberg

**Affiliations:** 1 Clinic for Orthopedics and Trauma Surgery, University Hospital Bonn, 53127 Bonn, Germany; 2 Department of Pediatric Surgery, University Children’s Hospital, 1004 Riga, Latvia; 3 Department of Pediatric Surgery, Riga Stradins University, 1007 Riga, Latvia; 4 Department of Spine Surgery, North Kurzeme Regional Hospital, 3601 Ventspils, Latvia; 5 Cantonal Psychiatric Hospital of Lucerne, 6000 Lucerne, Switzerland; 6 Department of Cardiovascular Medicine, Lady Davis Carmel Medical Center, 3436212 Haifa, Israel; 7 Department of Anesthesiology and Operative Intensive Care Medicine, Charité–Universitätsmedizin Berlin, Corporate Member of Freie Universität Berlin, Humboldt-Universität zu Berlin, and Berlin Institute of Health, 13353 Berlin, Germany; 8 Medical Faculty, University Hospital Essen, University Duisburg-Essen, 45147 Essen, Germany

**Keywords:** pediatric, scoliosis surgery, Latvia, operation time, hospital stay

## Abstract

*Background and Objectives:* There are currently no data available regarding pediatric scoliosis surgery in Latvia. The aim of this article is to present treatment specific variables, investigate their interrelation, and identify predictors for the length of stay after surgical pediatric scoliosis correction. *Materials and Methods:* This retrospective study included all surgical pediatric scoliosis corrections in Latvia for the years 2012 to 2016. Analyzed parameters were chosen to portray the patients’ demographics, pathology, as well as treatment specific variables. Descriptive, inferential, and linear regression statistics were calculated. *Results:* A total of 69 cases, 74% female and 26% male, were identified. The diagnostic subgroups consisted of 62% idiopathic (IDI) and 38% non-idiopathic (non-IDI) scoliosis cases. Non-IDI cases had significantly increased operation time, hospital stay, Cobb angle before surgery, and instrumented levels, while IDI cases showed significantly higher Cobb angle percentage correction. For all operated cases, the operation time and the hospital stay decreased significantly over the investigated time period. Early post-operative complications (PCs) occurred in 15.9% of the cases and were associated with increased hospital stay, instrumented levels, and Cobb angle before surgery. The linear regression analysis revealed that operation time and the presence of PCs were significant predictors for the length of the hospital stay. *Conclusions:* This is the first study to provide comprehensive insight into pediatric scoliosis surgery since its establishment in Latvia. Our regression model offers clinically applicable predictors and further underlines the significance of the operation length on the hospital stay. These results build the foundation for international comparison and facilitate improvement in the field.

## 1. Introduction

Spine deformity correction surgery is a major branch of pediatric surgery [1]. Since the first surgical spinal correction attempts and their detailed description in the year 1911 by Dr. Russell A. Hibbs, great technological progress has been achieved [2]. These advances have led to improved safety and significantly enhanced results of surgical scoliosis correction [3]. To date, surgery may halt curve progression and correct pathologic spinal curvature [4]. Among the pediatric spine deformities, idiopathic scoliosis is the most common [5,6,7]. Apart from idiopathic cases, etiologies of scoliosis are various and can be grouped into neuromuscular, congenital, and miscellaneous types—the latter including syndrome-related and secondary types [8] (Table 1). Congenital scoliosis is caused by vertebral anomalies leading to mechanical deviation of regular spine architecture, while neuromuscular scoliosis is caused by neurological (e.g., cerebral palsy, paralysis) or muscular disease (e.g., Duchenne muscular dystrophy). Lateral deviations of the spine in conjunction with conditions such as Marfan syndrome or neurofibromatosis are grouped under syndrome-related scoliosis [9,10]. In some cases, scoliosis can occur secondary to, for example, tumors, spinal cord abnormalities, or as a result of pain [11,12]. The term idiopathic is used if all possible causative disorders have been ruled out [13,14]. As the most common among the different types, it can be sub-classified according to the age of presentation: infantile (IIS; 0–3 years), juvenile (JIS; 3–10 years), and adolescent (AIS), which occurs by definition in children above age 10 until skeletal maturity [5,11].

In recent years, much effort has been made to characterize patients receiving spinal fusion surgery for non-idiopathic disease, such as neuromuscular [15,16,17], congenital [18], and syndromic [19] types, as well as for surgically treated idiopathic disease [20,21,22] at various centers around the world. While this treatment modality is currently regularly utilized in the Baltics, there is at present no literature reporting about scoliosis correction surgery for this region. In times of increasing relevance of machine learning, this paper presents a comprehensive analysis of pediatric scoliosis surgery in Latvia for the years 2012–2016, provides patient demographics, compares surgical data of the diagnostic subgroups, quantifies treatment-specific variables, and determines their relation to identify predictors for the length of the postoperative hospital stay. Our results allow for international comparison of scoliosis surgery and aim to facilitate future improvement.

## 2. Materials and Methods

### 2.1. Study Method, Data Acquisition, and Selection

Consent was obtained from the local universities ethical committee (Title: “Retrospective analysis of paediatric spine surgery at the University Children’s Hospital (Riga) from 2011 until 2016”, ID: 05, 27 October 2016). A retrospective analysis was conducted for all patients treated with scoliosis correction surgery in Latvia within the period from January 2012 until December 2016. This explorative study design included all possible cases, which made a priori power analysis not feasible. However, parameters and analysis were chosen based on previous studies. Data of scoliosis patients who received any form of spinal surgery within the period was retrieved from the digital medical record system of the Children’s Clinical University Hospital, Riga, the only medical center in Latvia that offers surgical scoliosis correction. The criterion for the final analysis was primary scoliosis correction within the period of interest. In concordance with previous studies, patients up to age 21 (≤21 years) were included to confine the analysis to the pediatric spectrum and minimize the potential influence of degenerative scoliotic changes at higher ages [23]. Cases with a postoperative stay (from the first day after the surgery) for more than 3 weeks were excluded, since these constituted complicated cases that required multiple follow up surgeries and were, as exceptions, not representative for the majority of cases. Since these constituted extreme outliers, they did not satisfy the assumption of the ensuing regression analysis. Of 82 identified cases, 9 received surgical treatment other than primary scoliosis correction, 2 exceeded the age limit, and 2 had a postoperative hospital stay longer than 21 days. Thus, 13 patients were excluded. The parameters analyzed were demographic data, diagnosis, admission and discharge date, main curve position and its direction, Cobb angle before and after the operation (accordingly within 6 months before and after the operation, obtained from the radiologic description), total number of vertebrae instrumented (as documented in the operation notes), total operation time (from incision to closure), and occurrence of early postoperative complications (defined in Table 2).

### 2.2. Statistical Analysis

Descriptive statistics, where appropriate, differences (Mann–Whitney U test, Chi-Square test, Kruskal–Wallis test), correlations (Spearman Correlation), and a linear multivariable regression analysis were calculated using a significance level of 95% (*p* < 0.05). The collected data were analyzed using IBM SPSS (Statistical Package for the Social Sciences).

## 3. Results

### 3.1. Included Subjects, Gender, Age, Type of Operation Diagnosis

A total of 69 operated scoliosis cases were enrolled, 74% female (n = 51) and 26% male (n = 18). Idiopathic cases comprised 62% of all cases, of which 52% were adolescent idiopathic scoliosis, 10% juvenile idiopathic scoliosis, and 0% infantile idiopathic scoliosis. The non-idiopathic group (38%) consisted of 25% neuromuscular, 7% syndromic, and 6% congenital cases. The mean age at surgery across all diagnostic groups was 14.6 ± 2.9 years (Table 3). The age at operation varied among idiopathic and non-idiopathic cases (*p* = 0.008, Mann–Whitney U).

All operative scoliosis corrections were achieved by spinal fusion surgery. Overall, 84.1% of the operations (n = 58) were performed by means of a posterior approach alone, 14.5% (n = 10) by a combination of anterior and posterior (360°) approach. The types of instrumentation used for the corrections were pedicle screw instrumentation (>80% of implants pedicle screws) in 72.5% (n = 50), hybrid instrumentation (hooks, wires, pedicle screws, combined approach) in 23.2% (n = 16), Shilla growth guidance in 2.9% (n = 2), and only anterior approach in 1.4% (n = 1). Luque rods, VEPTR (vertical expandable prosthetic titanium ribs), or growing rods have not yet been utilized in Latvia.

### 3.2. Curve Types and Direction

Across the whole patient population, 74% (n = 51) had a main curve convex to the right and 26% (n = 18) to the left. Among the neuromuscular patients were more cases of left-sided main curve (53.0%) than among the idiopathic patients (18.2%), (*p* = 0.019; Chi2 test).

The spine parts affected by the main curve were categorized as thoracic, thoracolumbar, and lumbar, as defined by the radiologic description. The overall frequencies were 50.7% (n = 35) thoracic, 46.4% (n = 32) thoracolumbar, and 2.9% (n = 2) lumbar. Isolated lumbar curves (n = 2) occurred only among neuromuscular patients. Comparing the frequencies of the most commonly occurring curve types, 65.1% (n = 28) among idiopathic patients had thoracic and 34.9% (n = 15) had thoracolumbar/lumbar curves, unlike neuromuscular patients, for whom 11.8% (n = 2) had thoracic and 88.2% (n = 15) had thoracolumbar/lumbar curves (*p* < 0.001, Pearson Chi-Square).

### 3.3. Idiopathic vs. Non-Idiopathic

Idiopathic cases (n = 43) had, compared to non-idiopathic cases (n = 26), a significantly decreased operation time, total number of vertebral instrumentation, hospital stay, and Cobb angle before and after the operation. The percentage decrease of the Cobb angle and the age of operation was significantly higher compared to the non-idiopathic group (Table 4). For the same variables, no differences were found within the idiopathic group between adolescent and juvenile cases, except for the age at operation (Table 3, *p* < 0.001). The gender distribution within the idiopathic group (86% female, 14% male) differed significantly compared to the non-idiopathic group (54% female, 46% male; *p* = 0.003, Pearson Chi-square).

### 3.4. Hospital Stay, Early Postoperative Complications, Operation Time

The average hospital stay across all diagnostic groups was 9.9 ± 2.6 days. Idiopathic scoliosis patients had the shortest mean hospital stay, about two days less than the non-idiopathic group (Table 4, *p* = 0.005). Early postoperative complications occurred in 15.9% (n = 11) of the cases (Table 2), and their incidence did not vary significantly among individual diagnostic groups (*p* = 0.208, Pearson Chi-square). The presence of PC was associated with a 3.2 days longer mean postoperative hospital stay (*p* = 0.001, Mann–Whitney U). There was no association between longer operation times and the incidence of early postoperative complications (*p* = 0.056), but larger Cobb angles before the operation (*p* = 0.006) and the total number of vertebrae instrumented (*p* = 0.010) were associated with higher incidences of PCs (Table 5).

**Figure 1:**
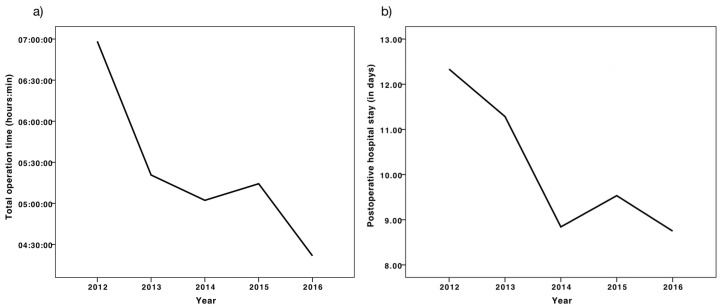
(**a**) Mean length of the surgery (hh:mm) between 2012–2016, (**b**) mean length of the hospital stay (in days) between 2012–2016.

The postoperative hospital stay across all diagnostic groups decreased significantly throughout the five year period (*p* = 0.002, Kruskal–Wallis test). The incidence of PCs did not influence this decline, since during the years 2015 and 2016, more PCs occurred (n = 6) than within 2012, 2013, and 2014 combined (n = 5). The mean hospital stay over the investigated period is depicted in Figure 1b

A similar decline was found for the operation time, which decreased during the investigated years (Kruskal–Wallis Test *p* = 0.013), as shown in Figure 1a. The temporal distribution of factors potentially influencing this decline, such as the use of the 360° surgery (*p* = 0.782, Pearson Chi-Square), the total number of vertebrae instrumented (*p* = 0.287), or the Cobb angle before the operation (*p* = 0.363, Kurskal–Wallis) did not vary significantly between the investigated years.

### 3.5. Correlations and Regression Analysis

Many of the analyzed parameters display inherently high degrees of collinearity, since their relationship is coined by direct causality. It is plausible that larger Cobb angles before the operation demand a larger extent of vertebral instrumentation (r = 0.440, *p* < 0.001) and consequently lead to longer operation times (r = 0.478, *p* < 0.001), as shown in Table A1. However, in addition to the obvious relations, the length of the hospital stay was strongly linked to the Cobb angle before the operation (r = 0.489, *p* < 0.001), the number of vertebrae instrumented (r = 0.526, *p* < 0.001), and total operation time (r = 0.614, *p* < 0.001), with the latter exhibiting the strongest interdependence and significance level. Further analysis revealed that, with exception for the hospital stay (*p* = 0.002) and the total operation time (*p* = 0.013), none of the variables included in the correlation analysis were significantly differently distributed within the five year investigated period (Kruskal–Wallis). Also, the relative sample size of the diagnostic groups did not vary significantly during this period and therefore did not influence this decline.

Setting the total length of the postoperative hospital stay (in days) as a dependent outcome, a linear multivariable regression analysis was performed to identify independent predictors of the hospital stay. Our analysis showed that the total operation time in hours (B = 1.0, 95% CI (0.7–1.3), *p* < 0.001) and the occurrence of early postoperative complications (B = 1.6, 95% CI (0.2–2.9), *p* = 0.024) with a constant coefficient (B = 2.8, 95% CI (0.9–4.7), *p* = 0.004) predict the length of the postoperative hospital stay (Table 6). The coefficient of determination was R2 = 0.488 and collinearity tolerance of xct = 0.86 satisfying. Using this model, the variables “total operation time” and “presence of early postoperative complications” were found to be independent predictors of 48.8% of the variance in the length of the postoperative hospital stay (*F*-value: 31.5, *p* < 0.001, ANOVA).

## 4. Discussion

### 4.1. Adolescent Idiopathic Scoliosis

Our results show that, among the different diagnostic subgroups, AIS corrections were the most common. This is in concert with previous investigations showing that most pediatric scoliosis correction surgeries are performed for AIS [20,21,24]. These patients were older at the operation with a mean of 16.0 ± 1.5 years in Latvia. In comparison, two hospitals in Atlanta described a mean age at operation of 14.4 ± 1.9 years and 14.7 ± 2.3 years for their AIS patient groups between 2006 and 2008 [20]. A similar study by Heller et al. in Kanas City, Missouri, which analyzed data of 95 AIS posterior fusion surgeries from 2011–2013, found a mean age at operation of 14.2 ± 1.8 years [25]. To partly explain the comparatively late operative intervention, it has to be kept in mind that there is currently no uniform screening program for the early detection of scoliosis in Latvia. However, even with early detection, it is often the patient’s preference to delay a surgical correction as long as possible. Although the clinical significance of an older age at the operation is not completely understood, there are reports stating that an earlier surgical intervention may increase the percentage correction rate of scoliotic curves [26].

As expected, the ratio of female patients for AIS correction outweighed the males—in Latvia by 86%—a number that reflects the asymmetry in incidence and is similar to the results from Missouri, where females made up 91.6% of the surgical AIS corrections [25]. Furthermore, Heller et al. reported a mean of 10.2 ± 2.1 fused vertebral levels similar to the data from Latvia with a mean of 9.7 ± 1.9, suggesting that the extent of vertebral instrumentation did not vary much among the two centers. Heller et al. also reported a mean operation time of 375 ± 72.7 min (06:15, hh:mm), which was longer compared to surgeries at our center with a mean of 290 ± 67 min (04:50).

### 4.2. Neuromuscular Scoliosis

The non-idiopathic patient group (n = 27) is subdivided into neuromuscular (n = 17), syndromic (n = 5), and congenital (n = 4) scoliosis patients. In Latvia, neuromuscular scoliosis patients who received surgical correction had a mean age at operation of 13.8 ± 3.8 years. One large study found a mean age of 12.8 years in the US [27], which is younger, but depending on the specific diagnosis, the age at operation may vary considerably for neuromuscular scoliosis correction. For example, Pesenti et al. reported a mean age at operation of 16.5 years for cerebral palsy patients and 13.9 years for Duchenne muscular dystrophy patients [17]. Our analysis of the subgroup of seven patients with cerebral palsy showed a mean age at operation of 15.4 ± 1.7 years. The variability of the age at operation in our patient population may be largely influenced by the heterogeneity of the underlying neuromuscular diseases in the investigated group.

In Latvia, the Cobb angle decreased for neuromuscular cases by a mean of 49.7 ± 21.1%, from a mean Cobb angle of 78.7 ± 20 degrees before the operation to 39.2 ± 19.4. A comparable study by Nordon et al. found at a mean age of 15.9 years and a mean percentage correction of 42.8% from a mean Cobb angle of 78.8 to 44.6 degrees. They also reported that patients stayed a mean average of 10 days at the hospital [28], which is slightly less than patients in Latvia with 11.8 ± 2.6 days. Although a direct comparison between these results is difficult, they may suggest that an operation at a younger age could lead to higher Cobb angle correction percentages and less hospitalization time for patients with neuromuscular scoliosis.

### 4.3. Total Operation Time and Length of the Hospital Stay

In 2012, pediatric scoliosis correction surgery had been established in Latvia. The continuous decrease in operation length may be explained by the gained experience from each consecutive operation. This learning curve has been reported in other studies, which showed that the number of previously operated surgeries had a greater effect on the operation length than, for example, the amount of vertebral levels fused [29]. Although there are many variables that influence the length of the surgery, such as the Cobb angle or the curve type, the experience operating surgeon has been shown to be most predictive [25]. Our results suggest that gained experience has led to decreasing durations of the surgical procedures. The postoperative hospital stay has been shown to be shortened accordingly. This phenomenon has been reported for spinal fusion surgeries as well as for other types of surgery [29,30,31,32]. Using our regression model, each additional hour of the operation increased the postoperative hospital stay by one day and the presence of PC by 1.6 days. Total operation time and hospital stay decreased significantly over the investigated period, while the incidence of early postoperative complications stayed constant. Although there are many factors influencing the length of hospital stay, the total operation time displays the strongest correlation with the length of the postoperative stay. This correlation predicts together with the presence of PC 48.8% of the variance of the postoperative stay in our model.

## 5. Conclusions

The present paper characterizes the patient population receiving primary scoliosis correction surgery in Latvia in the years from 2012–2016. It enables, for the first time, detailed analysis and comparison with other countries. Our regression model offers significant predictors and underlines, along with other studies, the influence of the operation time on the hospital stay. Overall, this study provides comprehensive insights into pediatric scoliosis surgery in Latvia, which has previously been undocumented and therefore not comparable with other cohorts. For this purpose, an explorative retrospective study design delivers valuable data. Nevertheless, conclusions drawn from a retrospective study are subjected to confounding and collinearity. They should be considered carefully and viewed within the context of current literature.

## Figures and Tables

**Table 1:** Classification of scoliosis—with courtesy of Choudhry MN et al. [5].

**Congenital:** Failure of formation, Failure of segmentation	**Idiopathic:** Infantile (0–3 years) Juvenile (3–10 years) Adolescent (10+ years until skeletal maturity)
**Neuromuscular:** **(1) Myopathic:** Arthrogryposis, Muscular dystrophy **(2) Neuropathic:** Upper Motor Neuron, Lower Motor Neuron, Dysautonomia	**Others:** Neurofibromatosis, Mesenchymal (Marfan’s, Ehler-Danlos), Traumatic, Tumors, Osteochondrodystrophies

**Table 2:** Categories and particular sums of early postoperative complications (PCs) of specific causes. PCs are defined as complication occurring within 2 weeks following the intervention. Intensive care unit (ICU), number (N).

Category	Specific Cause	N
General	Lung related complications (infection, embolism)	0
	Prolonged ICU stay (>2 days for posterior, >3 days for anterior-posterior approach)	2
	Development of pressure ulcer	1
	Anemia requiring blood transfusion	3
Wound related	Surgical wound complications (skin closure defects, infection)	1
Neurologic	Neurologic complications (neurological deficits)	1
	Urinary retention (requiring catheterization) and postoperative ileus	1
Instrumentation	Early revision surgery (problems with drainage, closure, infection, screw malposition)	2
Death		0

**Table 3:** Diagnostic groups with their respective median age at operation with standard deviation.

Diagnostic Groups	% (n)	Mean Age at Surgery	SD
All	100 (69)	14.6	2.9
Idiopathic scoliosis	62.3 (43)	15.4	2.1
Adolescent	52.2 (36)	16.0	1.5
Juvenile	10.1 (7)	11.9	1.1
Infantile	0	-	-
Non-idiopathic scoliosis	37.7 (26)	13.4	3.7
Neuromuscular *	24.6 (17)	13.8	3.8
Syndromic **	7.2 (5)	12.6	3.5
Congenital	5.8 (4)	12.8	4.1

* Neuromuscular cases subdivided into (n): cerebral palsy (7), spinal muscular atrophy (4), spinal bifida myelomenigocele (3), microcephaly with tetraparesis (1), infantile menigoencephalitis (1), agenesia of the corpus callosum (1); ** syndromic cases subdivided into (n): Marfan syndrome (3), neurofibromatosis (3).

**Table 4:** Differences between idiopathic and non-idiopathic scoliosis cases.

Variable	Idiopathic	(AIS)	Non-Idiopathic	(NEU)	*p*
Age at operation (in years)	15.4 ± 2.1	(16.0 ± 1.5)	13.4 ± 3.7	(13.8 ± 3.8)	** (**)
Total operation time (h:mm)	4:46 ± 1:05	(4:50 ± 1:07)	6:05 ± 1:45	(6:12 ± 1:39)	** (**)
N° of vertebrae instrumented	9.1 ± 1.8	(9.7 ± 1.9)	10.7 ± 3.7	(12.5 ± 2.7)	0.062 (***)
Postoperative hospital stay (in days)	9.2 ± 1.9	(9.2 ± 2.0)	11.2 ± 3.1	(11.8 ± 2.6)	** (**)
Cobb angle before (Cobb°)	55.8 ± 14.7	(56.3 ± 15.3)	75.1 ± 23.6	(78.7 ± 20.0)	** (***)
Cobb angle afterwards (Cobb°)	21.1 ± 9.0	(20.9 ± 9.2)	37.0 ± 18.4	(39.2 ± 19.4)	*** (***)
Percentage decrease (%)	62.0 ± 13.7	(62.6 ± 13.9)	49.7 ± 20.1	(49.7 ± 21.1)	* (*)

Differences (Mann–Whitney U test) between idiopathic n = 43 (AIS n = 36) and non-idiopathic n = 26 (NEU n = 17) scoliosis cases expressed as mean with standard deviation; significance level: * *p* < 0.05, ** *p* < 0.01, *** *p* < 0.001; *p*-values without parenthesis refer to the comparison of idiopathic vs non-idiopathic cases, *p*-values with parenthesis refer to the comparison of AIS vs. NEU; AIS: adolescent idiopathic scoliosis, NEU: neuromuscular scoliosis.

**Table 5:** Clinical parameters across all diagnostic groups.

Associations with PCs	Without PC	MC with PC	*p*-Value
Age at operation (in years)	14.5 ± 2.9	+0.9	0.818
Total operation time (hh:mm)	5:01 ± 1:09	+1:33	0.056
N° of vertebrae instrumented	9.7 ± 2.6	+2.4	*
Postoperative hospital stay (in days)	9.4 ± 2.3	+3.1	**
Cobb angle before (Cobb°)	59.7 ± 17.6	+21.5	**
Cobb angle afterwards (Cobb°)	25.0 ± 12.0	+13.2	0.158
Percentage decrease (%)	57.6 ± 17.4	−1.5	0.737

Parameters across all diagnostic groups and their mean change (MC) in case of early post-operative complications (PCs) are depicted (Mann–Whitney Test); significance level: *p < 0.05, **p < 0.01.

**Table 6:** Multivariable linear regression analysis for the length of the dependent variable postoperative hospital stay. Model N = 95, *F*-value 31.5, *p* < 0.001, R2 = 0.488.

Variables	Regression Coefficient	Standardized Coefficient	*p*-Value	95% CI
Total operation time (hours)	1.01	0.586	<0.001	0.683–1.337
Presence of PC	1.56	0.220	0.024	0.213–2.898

## Appendix A

**Table A1:** Spearman’s correlation of the analyzed numerical variables.

Variable	AAO	TOT	TVI	POHS	CAB	CAA	PC
Age at operation (AAO)	1						
Total operation time (TOT)	−0.139	1					
Total vertebral instrumentation (TVI)	0.073	0.410 (**)	1				
Postoperative hospital stay (POHS)	−0.130	0.614 (**)	0.526 (**)	1			
Cobb angle before (CAB)	−0.198	0.478 (**)	0.440 (**)	0.489 (**)	1		
Cobb angle afterwards (CAA)	−0.148	0.409 (**)	0.243 (*)	0.327 (**)	0.530 (**)	1	
Percentage correction (%C)	0.060	−0.166	−0.092	−0.081	−0.036	0.837 (**)	1

Age at operation (AAO), Total operation time (TOT), Total vertebral instrumentation (TVI), Postoperative hospital stay (POHS), Cobb angle before (CAB), Cobb angle afterwards (CAA), Percentage correction (%C); significance level: **p* < 0.05, ***p* < 0.01.
